# Footprints of past mining in Alaska (USA) derived from high-resolution satellite imagery

**DOI:** 10.1038/s41597-025-05039-z

**Published:** 2025-04-25

**Authors:** Adrian Bender

**Affiliations:** https://ror.org/05ehhzx21 U.S. Geological Survey Alaska Science Center, 4210 University Drive, Anchorage, AK 99508 USA

**Keywords:** Environmental impact, Environmental economics

## Abstract

Mapping the land area used for mining in the past is essential for guiding the remediation of affected landscapes and assessing the resource potential of related waste products. Despite significant recent progress delineating footprints of active and inactive mining globally, the known inventory of such mine lands remains incomplete. Here, I describe a new map dataset of footprints of land surface disturbance and waste at sites of past mining in Alaska (USA) based on visual interpretation of satellite imagery. This dataset maps 6–14 times the area of previous regional and global mine footprint maps in Alaska and is the first in the region to explicitly delineate mine waste landforms (e.g., tailings piles). The data are publicly available from the U.S. Geological Survey under a “no rights reserved” Creative Commons (CC0) license agreement.

## Background & Summary

Despite recent progress^1–3^, the area of land use by mining worldwide remains under-documented^4^. This is attributable in part to inadequate reporting of contemporary and ongoing mining, and to an incomplete inventory of known historical (i.e., presently inactive) mine land footprints^4^. Knowledge of the location and spatial extent of legacy mine land is particularly important for (1) remediation of mining-driven landscape, waterway, and ecosystem disturbances^5^ that can persist over centennial timescales even at relict sites (e.g., references^6,7^), and (2) assessment of the mineral resource potential associated with unrecovered commodities in related mine or process wastes (e.g., tailings or rock) abandoned during the past century^8–10^.

Most mine footprint maps are the product of visual image interpretation and manual delineation of representative polygons^1–4,11,12^, but artificial intelligence techniques are also emerging^13–15^. These efforts are aided by the wide availability of high-resolution satellite imagery, but hindered by limited point datasets that archive the locations and statuses of mining activities. Standard & Poor’s proprietary SNL metals and mining database^16^, for example, is commonly used to interrogate the global mining sector^4^. The SNL database provides the basis for an evolving global mine footprint dataset that is based on geomorphic evidence of mining identified on satellite imagery within 10-kilometre radiuses of active and inactive SNL mine locations^2,3^. A follow-up study expanded the number of mine footprints from 44,929 polygon features (101,583 km^2^ total)^3^ to 74,548 polygons (65,585.4 km^2^ total) by incorporating mine sites archived in scientific, government, and industry literature as well as new sites identified during mapping^1^. The ~65% increase in the number of mapped polygons highlights both the incompleteness of the SNL point database and a difference between these studies’ map criteria and scale. For example, despite incorporating more individual sites, the finer scale of the updated dataset led to a ~35% decrease in total mapped area^1^. The global mining footprint^1^ provides an important snapshot of global land area used by mining, including both active and inactive mine sites from various sources. Scant coverage in certain prominent mining regions including Alaska (USA), however, demonstrate that the global mining footprint inventory remains incomplete.

Here, I present a new dataset that delineates footprints of disturbed ground and waste associated with past mining in Alaska^17^. Alaska occupies ~7% of North America by land area and has a history of continuous mining since at least the late 1800s CE (e.g., reference^18^). Point locations of historical mining in Alaska are well-documented by the U.S. Geological Survey Alaska Resource Data File (ARDF)^19^ point dataset, but the areal extent of past mine land use remained largely unconstrained until now. To map footprints of past mining in Alaska, I used a process similar to that used to generate the global mine footprint datasets^1–4,11,12^. Specifically, I used a subset of ARDF^19^ points to guide visual interpretation and manual delineation of representative polygons on high-resolution satellite imagery. I developed this dataset to locate, quantify, and inventory areas of (1) un-remediated disturbed ground resulting from past mining, and (2) waste products (*sensu* reference^8^) of past mining that occur at the surface and may contain un-recovered mineral resources. The footprint data may help inform Alaska land managers of the inherited impact of past mining on the landscape and can be used as a tool to remotely prospect the re-mining potential of waste at legacy mine sites.

## Methods

**Fig. 1:**
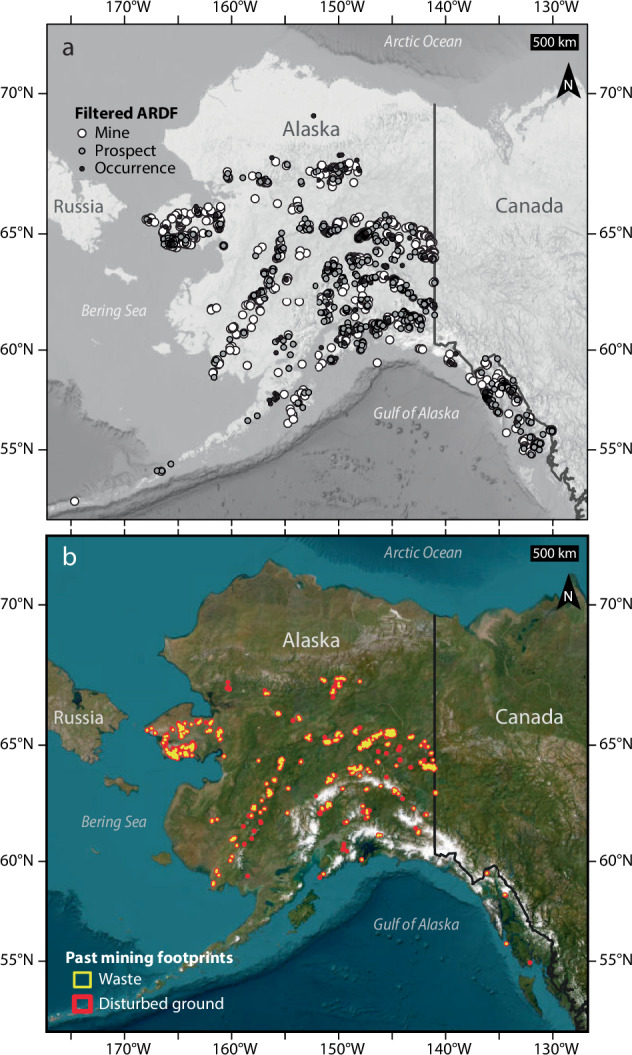
Sites and footprints of past mine production in Alaska. (**a**) Subset of Alaska Resource Data File (ARDF)^19^ sites filtered by activity and production (n = 2,125) and used to guide footprint mapping of the dataset described in this paper. Base is the 2023 General Bathymetric Chart of the Oceans greyscale basemap (https://noaa.hub.arcgis.com/maps/noaa::gebco-grayscale-basemap-noaa-ncei-visualization/about). (**b**) Footprints of past mining contained in this dataset^17^. Disturbed ground (red, n = 748) and waste (yellow, n = 551) plotted over World Imagery^20^.

**Fig. 2:**
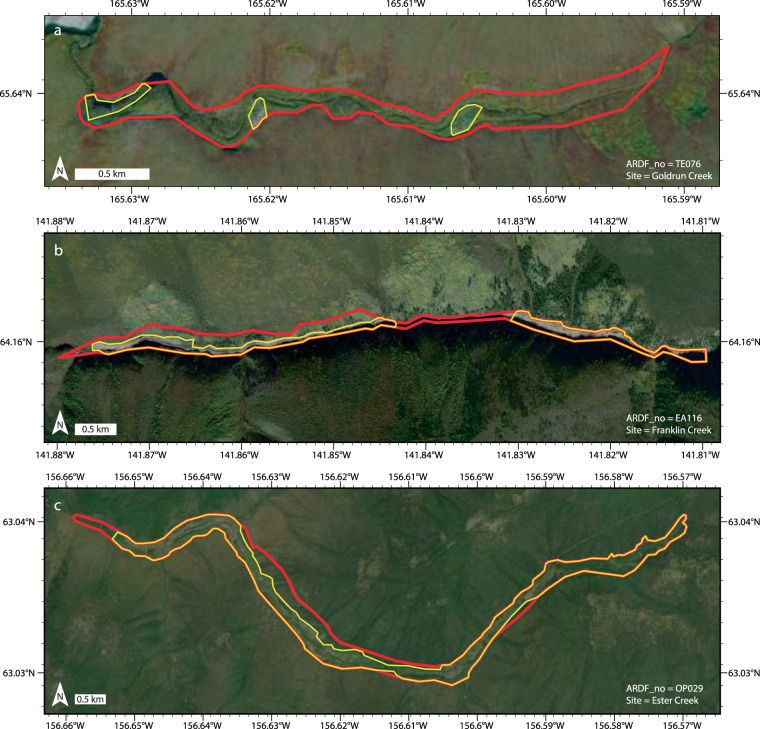
Examples of disturbed ground and waste footprints^17^. Shown are the (**a**) 16^th^, (**b**) median, and (**a**) 84^th^ percentile sites by waste footprint area. Map symbols after Fig. 1—red lines show disturbed ground footprints, yellow lines show waste footprints.

The purpose of this dataset is to map footprints of disturbed ground and mine waste at sites in Alaska where past mine production occurred but is no longer underway. I defined these sites by querying the Alaska Resource Data File (ARDF)^19^, a U.S. Geological Survey point dataset that is the authoritative record of mines, prospects, and mineral occurrences in Alaska (Fig. 1a). The queried ARDF sites guided identification and delineation of mine-related disturbed ground and waste on high-resolution satellite imagery (Figs. 1b, 2a–c). In this section, I describe the process steps used to produce the dataset, all of which were performed in ArcGIS Pro 3.2.2.

### Filtering the ARDF

I filtered a subset of ARDF records based on present activity and past commodity production by querying the attributes ‘site_status’ and ‘production’ (Fig. 1a). The ‘site_status’ field describes contemporary mining or exploration activity at a given ARDF site with values of ‘Active’, ‘Active?’, ‘Probably Inactive’, ‘Inactive’, and ‘Undetermined’. The ‘production’ field describes the existence and relative size of an ARDF site’s total mineral commodity production with values of ‘Yes; large’, ‘Yes; medium’, ‘Yes; small’, ‘Undetermined’, and ‘None’. I used the definition query “site_status <>‘Active’ And production <> ‘None’” to select ARDF sites that (1) are unlikely to presently be experiencing active mining, and (2) have or may have produced mineral commodities in the past. This query yielded a sub-set of 2,125 ARDF sites that I visually inspected for the presence or absence of disturbed ground on satellite imagery.

### Disturbed ground footprints

I visually inspected the variable-resolution World Imagery^20^ Maxar satellite product for the presence or absence of disturbed ground within a 10 km radius of each of the 2,125 definition-queried ARDF sites. The 10 km search radius is commonly applied in the literature (e.g., reference^3^), and in this case reflects a practical upper limit based on reconnaissance visual inspection of World Imagery^20^ around ARDF point locations with coordinate accuracies that may exceed 1 km. I accessed World Imagery, an ArcGIS Online map service providing global seamless and cloudless satellite and aerial imagery at ≥ 37 cm resolution acquired within 3–5 years of access date ^20^, between March and December 2023. I defined ‘disturbed ground’ as apparently unnatural (i.e., human-caused) physical disruptions to the Earth’s surface that may include discrete waste piles, ponds, dams, trenches, roads, scarred vegetation, or similar features that are distinct from the adjacent undisturbed landscape. In forested regions such as coastal southeast Alaska, dense vegetation may obscure disturbed areas from recognition in World Imagery.

Visual inspection of the World Imagery at a dynamic viewing scale of ≤1:20,000 revealed disturbed ground at 748 of the 2,125 filtered ARDF sites (Fig. 1b). At each of the 748 sites I digitized (≤1:20,000 scale) polygons that delineate the footprint perimeter of visually identified disturbed ground in a one-to-one relationship with the corresponding filtered ARDF record (Fig. 2). Consistent with prior approaches to inventorying mined lands at a global scale^2,3^, the footprints may encompass ground that is not directly disturbed but is instead fragmented by adjacent mining or mineral exploration. I attributed the disturbed ground footprints with ‘Site’ and ‘ARDF_no’ fields that report the site name and unique site number of the related ARDF record; each polygon or multi-part polygon represents disturbed ground at a unique and non-repeating site by ‘ARDF_no’, but the ‘Site’ field contains repeated entries reflecting non-unique site names. In cases where the ARDF divides a single site into two or more across map quadrangle boundaries, I assign a single ‘ARDF_no’ according to the quadrangle in which the largest disturbed area is mapped. I calculated footprint surface area in square kilometres in the field ‘area_km’ and exported the footprints as a shapefile. The field ‘ARDF_no’ can be used to join or relate the disturbance footprint polygon shapefile and the ARDF point shapefile.

### Waste footprints

I refined the 748 disturbed ground footprints into an additional polygon shapefile dataset of above-ground mine waste footprints (Figs. 1b, 2). At a dynamic scale of ≤1:20,000, I visually re-checked each of the 748 disturbance footprints for the presence or absence of above-ground mine waste landforms such as placer tailings piles on ≥37 cm-resolution World Imagery^20^ (accessed between December 2023 and April 2024). This check identified distinct mine waste landforms at 551 of the 748 disturbance footprints. I was unable to visually identify mine waste in 218 of the 748 disturbed ground footprints (Fig. 1b).

I digitized (≤1:20,000 scale) polygons delineating the perimeters of mine waste landforms within each of the 551 disturbance footprints on the World Imagery (Fig. 1b). The waste footprints occur entirely within the bounds of corresponding disturbed ground footprints and include multi-part polygons (e.g., Fig. 2). I attributed the waste footprints with “Site” and “ARDF_no” information from the disturbance footprints via spatial join, and calculated surface area for the mapped extent of waste at each site in square kilometres in the field ‘area_km’. In cases where the ARDF divides a single site into two or more across map quadrangle boundaries, I assigned a single ‘ARDF_no’ according to the quadrangle in which I mapped the largest waste area. As with the disturbance footprint shapefile, the field ‘ARDF_no’ can be used to join or relate the waste footprint polygon shapefile to the ARDF point shapefile.

## Data Records

**Fig. 3:**
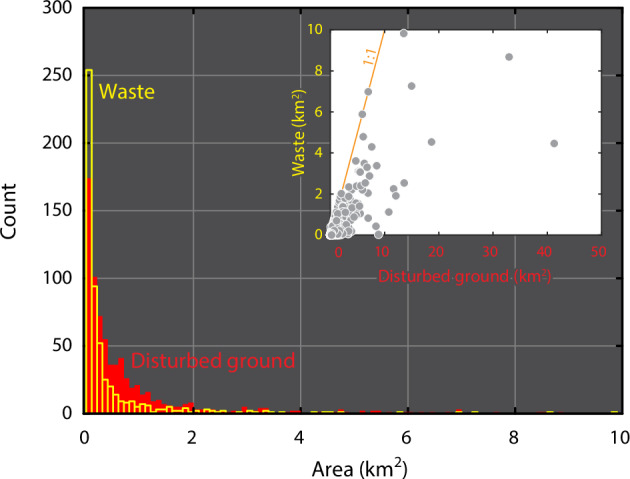
Area distribution of disturbed ground and waste footprints^17^. Histogram of disturbed ground (red, n = 748) and waste (yellow, n = 551) footprint areas, truncated at 10 km^2^. Bin width is 0.1 km^2^. Inset plots waste footprints against corresponding disturbed ground footprints by area (n = 551).

This dataset^17^ is the result of visual interpretation of ≥37 cm-resolution World Imagery^20^ within 10 km of filtered ARDF point localities at a dynamic scale no coarser than 1:20,000, and comprises two polygon shapefiles of disturbed ground (n = 748) and waste (n = 551) footprints at sites of past mining in Alaska (Fig. 1b). The waste footprints are mapped entirely within corresponding disturbed ground footprints. All footprints are attributed with the fields “Site”, “ARDF_no”, and “area_km”, that provide footprint site names and unique identifiers derived from the ARDF point dataset^19^, and footprint area in square km, respectively. The 748 unique disturbed ground footprints encompass a total area of 734.8 km^2^; the smallest footprint is 0.3 × 10^−3^ km^2^, the median is 0.3 km^2^, and the largest is 41.3 km^2^ (Fig. 3a). The 551 unique waste footprints encompass a total area of 239.4 km^2^; the smallest footprint is 0.3 × 10^−3^ km^2^, the median is 0.1 km^2^, and the largest is 9.8 km^2^ (Fig. 3). The two files of this dataset collectively provide detailed information about landscape impacts of past mining that expand on contributions of prior map datasets, and may be useful for interrogating legacy mine waste deposits as potential future mineral resources. The data are publicly served by the U.S. Geological Survey under a “no rights reserved” CC0 license agreement.

## Technical Validation

The disturbed ground and waste footprints of this dataset^17^ were delineated by a trained geomorphic mapper on high-resolution satellite imagery (≥37 cm) at a scale not coarser than 1:20,000. In this section I describe this dataset’s comparability to prior Alaska mine footprint maps^1–3,21^, and discuss known sources of confidence and uncertainty. I also describe the technical review that these map data underwent in accord with procedures standard to the publication of U.S. Geological Survey data releases.

### Comparison with prior Alaska mine footprint datasets

This dataset^17^ improves the resolution, accuracy, and spatial coverage of known historical land use by mining in Alaska. For example, a recent effort to inventory global mining land use areas mapped 34 polygons of disturbed ground at 16 active mines in Alaska (54.6 km^2^ total) on satellite imagery^2^, updated in a follow-up study to 86 mine footprints covering an unspecified number of individual mine sites (112.1 km^2^ total)^3^. In the updated global dataset^3^, 42 of the 86 polygons overlap footprints in the present dataset^17^. Visual inspection of World Imagery^20^ indicates that the 44 non-overlapping polygons represent active mines as well as non-mine features including highway gravel pits, a Fairbanks aggregate plant, and the Palmer Correctional Center (a prison facility). Another recent global mine footprint dataset delineated 78 Alaska mine footprints representing an unspecified number of individual mine sites (79.8 km^2^ total)^1^. Of these 78 footprints, 42 intersect polygons in the presently described dataset^17^. Visual inspection of World Imagery^20^ indicates that the remaining 36 footprints cover active mines and non-mine features such as highway gravel pits and at least one natural fluvial cutbank^3^.

**Fig. 4:**
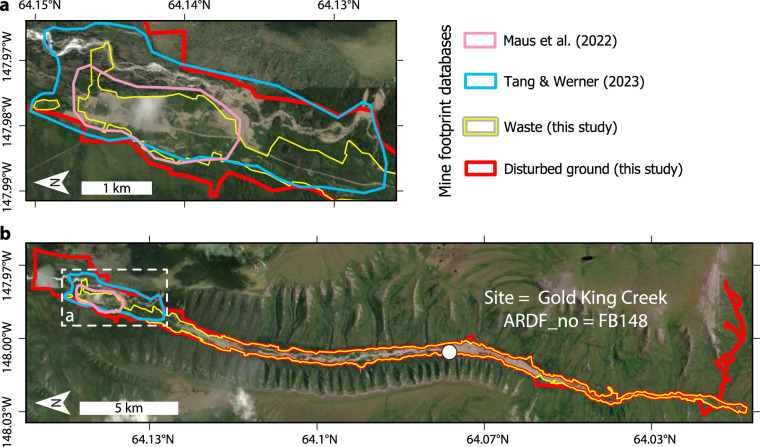
One of 24 instances of overlap between mine footprints in the global datasets of Maus *et al*.^3^, Tang & Werner^1^, and the dataset described in this study^17^. (**a**) Detail view of the mine footprints delineated in reference^3^ and reference^1^. (**b**) Overview of the site depicting the full extent of waste and disturbed ground footprints delineated in this study^17^. White dashed box marks the location of panel a. White dot marks the location of the ARDF record^19^.

Overlap between footprints in both global databases^1,3^ and the presently described dataset^17^ all overlap in 24 instances across 12 unique mine sites (e.g., Fig. 4a,b). The global datasets portray up to three polygons per site while the present dataset maps waste and disturbed ground footprints unique to each mine site. Where overlap occurs, polygons of the global datasets tend to cover smaller areas at a resolution that appears coarser than the ≤1:20,000 map scale of the presently described dataset^17^. Instances of polygons absent from the presently described dataset^17^ but present in the global datasets (44 in reference^3^, 36 in reference^1^) likely reflect map criteria, mine location data, and imagery that differ from those I used to produce the dataset described in this paper^17^. For example, the ARDF^19^ provided mine location data for the present dataset^17^ but was not used in development of the global datasets^1,3^. The ARDF contains >7,500 point locations for active and historical mining activity in Alaska; three orders of magnitude more locations than the 78–86 polygons depicting Alaska mine footprints in the global datasets^1,3^. Regional mine location datasets such as the ARDF therefore provide useful data for increasing the completeness of the known global mine footprint.

A recent regional study mapped 381 unique footprints of mining at sites in Alaska (477.2 km^2^ total) at unspecified scale on a variety of base data^21^. I refer to this University of Alaska Historic Mining Footprint dataset as the UAHMF^21^. Like the presently described dataset, the UAHMF relied at least in part on the ARDF to locate mine sites. It is not clear, however, what criteria were applied to the UAHMF for inclusion or exclusion of sites to be considered for mapping^21^. The UAHMF contains footprints of past mining that lump mine waste and disturbed ground at sites internally listed as active (n = 261) and inactive (n = 120). Spatially joining the 120 inactive-site UAHMF footprints to the disturbed ground footprints described in this paper via largest overlap within a 1-km radius yields 92 footprints (total UAHMF area = 113.8 km^2^).

**Fig. 5:**
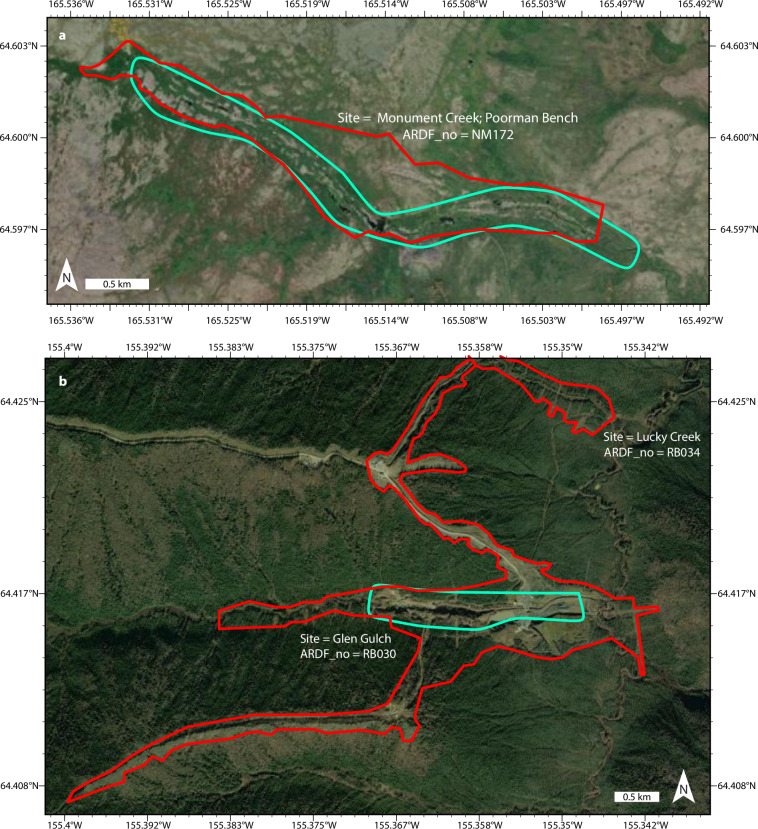
Example comparisons of disturbed ground footprints in this dataset^17^ (red) where the spatial relationship to UAHMF^21^ (cyan) polygons is one-to-one. (**a**) One-to-one spatial relationship where the footprints of this dataset accord reasonably well with the overlapping UAHMF polygon. (**b**) One-to-one spatial relationship where the footprints of this dataset disagree with the extent of overlapping UAHMF polygon. No UAHMF polygon overlaps the Lucky Creek disturbed ground footprint in the northwest corner of the map.

**Fig. 6:**
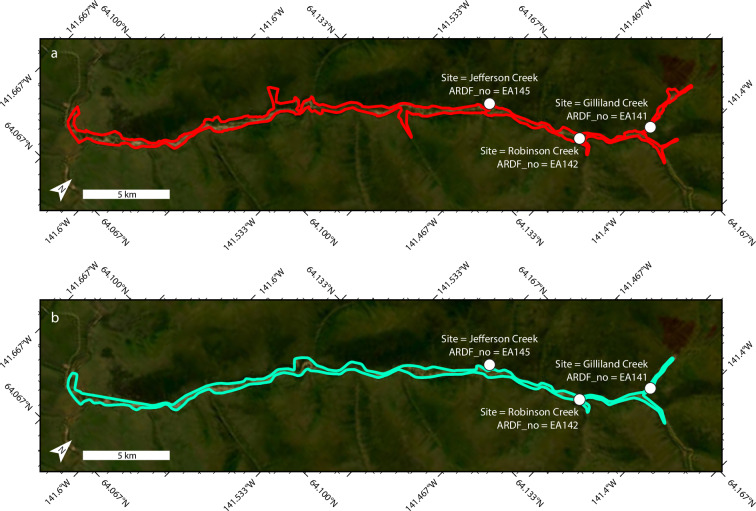
Example of a many-to-one relationship between footprints of the present dataset^17^ and the UAHMF^21^. (**a**) Three unique contiguous disturbed ground footprints (red) that represent the three ARDF^19^ localities (black dots, labelled with white text) from the present dataset^17^, represented by (**b**) a single polygon in the UAHMF dataset (cyan).

**Fig. 7:**
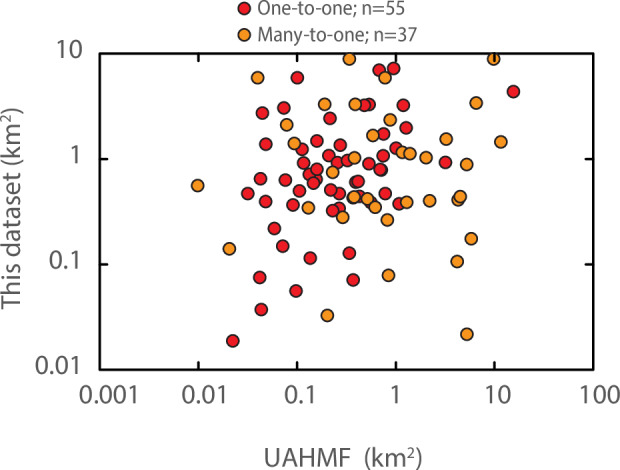
Disturbed ground footprints of this dataset^17^ plotted by area against co-located UAHMF^21^ polygons (largest overlapping within 1 km).

Comparison of the 92 spatially overlapping footprints reveals discrepancies between the UAHMF and the presently described dataset (Figs. 4, 5). Discrepancies may be related to differences in mapping scale and/or criteria between studies. Visual inspection on World Imagery implies that the unspecified scale of the UAHMF is generally coarser than the ≤1:20,000 acquisition scale of the presently described dataset^17^ (e.g., Figs. 4, 5). In 55 of the 92 spatially joined footprints, the relationship between the UAHMF and presently described dataset is one-to-one (Fig. 5). In the remaining 37 one-to-many cases, a single UAHMF footprint may cover an area that is occupied by multiple contiguous unique disturbed ground footprints in this dataset that are associated with unique adjacent ARDF localities (Fig. 6). The discrepancies might be expected to reflect a tendency for one dataset to contain systematically larger or smaller footprints, but no such trend is evident (Fig. 7).

Visual inspection of World Imagery^20^ indicates that the 28 non-overlapping inactive-site UAHMF footprints comprise (a) 19 footprints that are either not mines (e.g., gravel pits or telecommunications pads) or are not within 10 km of an inactive ARDF locality and thus not considered in the present dataset, (b) seven footprints that are evidently active on World Imagery (i.e., equipment and ‘fresh’ excavation present) and not within 10 km of an inactive ARDF locality, and (c) two footprints totalling 4 square km that depict apparent inactive sites of past mining but are not within 10 km of an inactive ARDF locality. Hence, I suggest that these instances of polygons absent from the presently described dataset reflect map criteria and/or imagery used to generate the UAHMF that differ from those I applied to produce the dataset described in this paper^17^.

### Completeness, consistency, and accuracy

This dataset represents visually identifiable evidence for past mining production (i.e., waste piles, trenches, disturbed ground and/or vegetation) at ARDF sites by the definition query “site_status <> ‘Active’ And production <> ‘None’”. Within this scope, the dataset’s completeness is subject to interpretational limitations of the ≥37 cm-resolution World Imagery^20^ base image and the potential for differences in image interpretation between the author and other mappers. The dataset cannot be construed as a complete record of the footprint of past mining in Alaska because, at a minimum, the ARDF dataset that guided the footprint mapping provides an authoritative yet likely incomplete record of mining activities and locations in Alaska^19^.

This dataset^17^ therefore represents a minimum land area affected by mining in Alaska in terms of both disturbed ground and waste. For example, not all ground disturbance or above-ground waste may be visually identifiable on the World Imagery. The ARDF may not provide a complete record of historical mining given unreliable reporting of related land use over the past century^4^. Accordingly, two examples of inactive mine footprints are documented in the UAHMF dataset but not here; these footprints are not associated with ARDF records and are therefore exempt from the scope of mapping for this dataset. Additionally, World Imagery^20^ showed equivocal evidence of possible contemporary reoccupation of some sites in the form of excavation equipment or apparent ‘fresh’ vehicle tracks and/or excavation, so some uncertain fraction of the mapped sites may now be experiencing active footprint growth. Hence, this dataset provides a minimum estimate of ground disturbance and waste associated with past mining in Alaska, including rare sites that may have experienced contemporary mining or exploration activity.

In contrast to these sources of uncertainty, ARDF descriptions of site locations and workings provided useful information to corroborate the scale, presence, or absence of disturbed ground and waste visualized on the World Imagery during mapping. The map datasets contain no duplicate records by the unique site identifier ‘ARDF_no’, but the disturbed ground and waste footprint polygon shapefiles contain 88% and 89% unique ‘Site’ entries, respectively. The 11–12% non-unique ‘Site’ entries reflect usage of the same site name at different locations across the state of Alaska. For example, three unique sites called “American Creek” with distinct locations and values of ‘ARDF_no’ occur in the waste footprint file.

Footprint locations are consistent with the locations of corresponding ARDF points (i.e., one-to-one relationships on ‘ARDF_no’) within a 10 km radius. Mapped boundaries between contiguous footprint polygons were located based on either (1) discrete features such as roads, dams, and tributary junctions, or (2) interpreted differences in the degree or age of land use based, for example, on vegetation coverage and/or freshness of excavation. In both cases, such boundaries are interpretive and may not be accurately located. Finally, waste footprints are completely contained by their corresponding disturbed ground footprints by spatial extent (Fig. 2a–c). Calculated surface areas for waste footprints therefore do not exceed surface areas of disturbed ground footprints (e.g., Fig. 3 inset).

At the maximum 1:20,000 scale of mapping, the horizontal positional accuracy of the mapping is expected to be within meters, consistent with the resolution of the World Imagery^20^ on which the features were mapped. The footprints contain no vertical position information. I accessed the World Imagery to conduct mapping between March 2023 and April 2024; because this product images the landscape within 3–5 years prior to access date, future changes to the sites may result in inconsistencies between the present mapping and the imaged landscape.

### Technical review

Two trained image interpreters independently reviewed the map data as part of the standard U.S. Geological Survey internal review preceding publication. The reviewers cross-referenced the two footprint shapefiles against the filtered ARDF, and systematically checked the mapped footprints over the World Imagery to provide expert feedback where interpretations differed. Issues and potential errors revealed during the reviews were reconciled prior to publication of the data release^17^ and this paper. Additionally, two journal-assigned subject matter experts provided technical reviews of the dataset during review of this paper.

## Usage Notes

In this section I discuss several potential use cases for the presently described dataset^17^, which can be accessed online at 10.5066/P1UQYBDV and used without restriction under a CC0 license.

### Querying waste deposits as potential resources

Joining or relating the footprints to the ARDF on the field ‘ARDF_no’ enables exploration of site properties, including the deposit types and mineral commodities mined or explored at each footprint. For example, at ARDF sites associated with the 551 unique waste footprints, filtering the dataset for “Deposit_model” values containing the text “placer” implies that placer deposits account for 88% of mined deposits where I mapped visible waste.

**Fig. 8:**
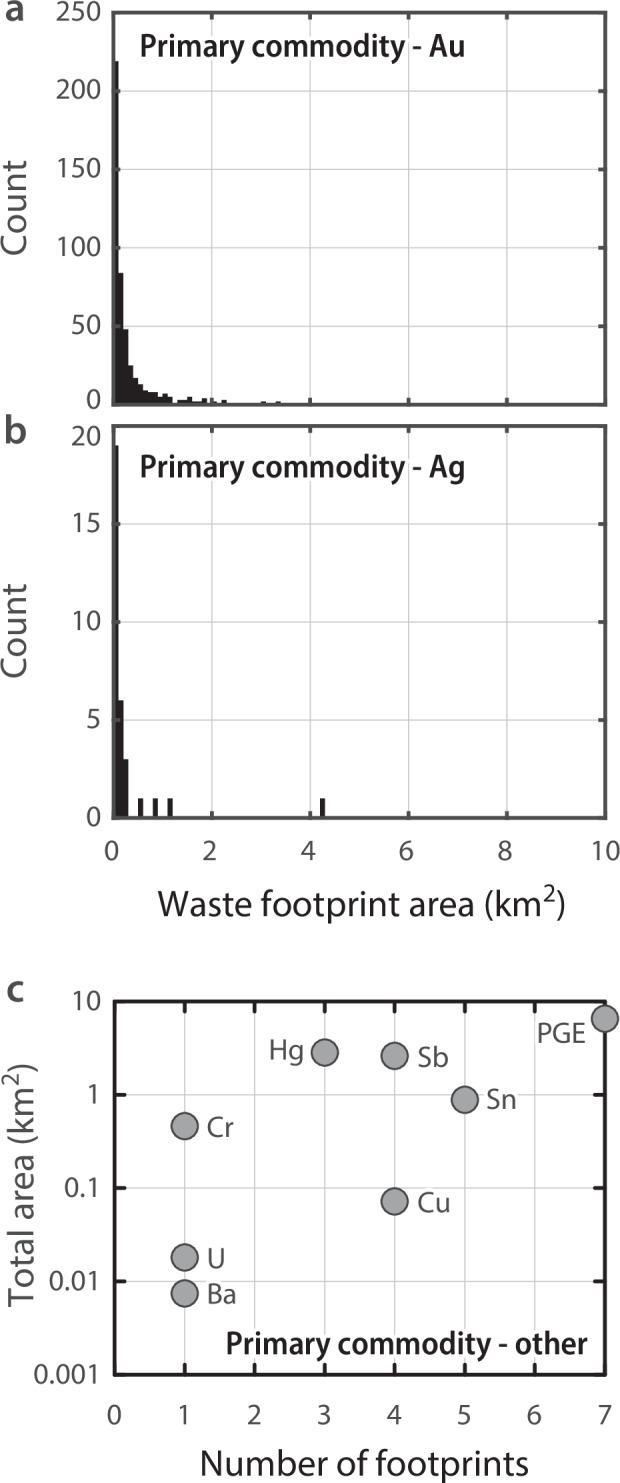
Distribution of waste footprint^17^ areas by ARDF^19^ first-listed main commodity. Histograms of footprints with primary commodities of (**a**) gold (n = 493), (**b**) silver (n = 32). (**c**) Scatter plot of the number of mine waste footprints versus total area of mine waste footprints with primary commodities other than gold or silver as labelled (n = 26). PGE—platinum group elements.

Of the 551 waste footprints, the ARDF lists ‘main commodities’ of gold at 493 sites and silver at 32 sites with areas totalling 214.7 km^2^ and 8.7 km^2^, respectively (Fig. 8a,b). The remaining 26 footprint sites have primary commodities that include platinum group elements (PGE, n = 7; 6.6 km^2^ total), tin (n = 5; 0.9 km^2^ total), antimony (n = 4; 2.6 km^2^ total), copper (n = 4; 0.1 km^2^ total), mercury (n = 3; 2.8 km^2^ total), and single instances of chromium (0.5 km^2^), uranium (<0.1 km^2^), and barium (<0.1 km^2^) (Fig. 8c). The ARDF also lists non-primary commodities associated with each site. Given deposit area-volume proportionality, this information could be used to target legacy mine waste deposits by size for potential reprocessing and commodity recovery by modern methods^8,22–25^. Further, multispectral data could be used to remotely approximate the composition of mapped mine waste^26^.

### Baseline applications

This dataset^17^ represents the footprint of past mine production in Alaska (~2019–2024 CE) where ARDF records exist^19^, and therefore provides a baseline for other approaches that might improve the spatial and temporal completeness of Alaska’s historical mine footprint. For example, this dataset could be used to train machine learning models to detect and map signatures of waste and disturbed ground associated with past mining in satellite imagery or in lidar derivatives as and when such data become available^27,28^. A machine learning approach has the potential to increase the completeness of the known historical mining footprint in Alaska. Additionally, changes over time to the total footprint of land use by mining in Alaska could be estimated by comparing the polygons of this dataset^17^ with legacy imagery, and future changes could be tracked by periodically revising the polygons over new satellite imagery.

## Data Availability

No custom code was used in the development of this dataset.
